# Bridging Discoveries and Treatments: The New Landscape of Breast Cancer Research

**DOI:** 10.3390/life14060747

**Published:** 2024-06-12

**Authors:** Taobo Hu, Lei Wang, Riccardo Autelli, Mengping Long

**Affiliations:** 1 Department of Breast Surgery, Peking University People’s Hospital, Beijing 100033, China; 2 International Cancer Center, Shenzhen University, Shenzhen 518060, China; 3 Department of Clinical and Biological Sciences, University of Turin, 10125 Turin, Italy; 4 Department of Pathology, Peking University Cancer Hospital, Beijing 100191, China

Welcome to our Special Issue, “Advances in Breast Cancer Research and Treatment” of *Life*, where we have embarked on a comprehensive exploration of groundbreaking studies that advance our understanding and management of breast cancer. Each paper contributes uniquely to the evolving landscape of breast cancer research, ranging from the clinicopathological characteristics of breast carcinoma with neuroendocrine features [1], the identification of hub genes in the context of non-alcoholic fatty liver disease and triple-negative breast cancer [2], to advancements in diagnostic techniques [3], and the exploration of immunotherapy biomarkers [4]. Some of the topics covered include innovative methods for breast cancer classification combining transfer learning and attention mechanisms [5] and the role of diffusion-weighted imaging in breast cancer diagnosis among young patients [6]. The collective insights presented here not only underscore the complexity of this disease but also highlight the promising pathways toward more effective treatments and improved patient outcomes.

## 1. Highlights from This Special Issue

### 1.1. Application of Deep Learning in Breast Cancer Pathology Image Classification

The article “Improved Breast Cancer Classification through Combining Transfer Learning and Attention Mechanism” introduces a novel approach that enhances the accuracy and interpretability of breast cancer histopathological image classification [5]. This method utilizes modified pre-trained Convolutional Neural Network (CNN) models and attention mechanisms to emphasize localized features and enable accurate discrimination in complex cases.

### 1.2. The Use of Diffusion-Weighted Imaging (DWI) in Young Breast Cancer Patients

“The Role of Diffusion-Weighted Imaging Based on Maximum-Intensity Projection in Young Patients with Marked Background Parenchymal Enhancement on Contrast-Enhanced Breast MRI” explores the application of DWI, particularly in young patients with significant background parenchymal enhancement (BPE) on contrast-enhanced MRI (CE-MRI) [6]. This study found that DWI outperforms CE-MRI in terms of lesion detection.

### 1.3. Association between Non-Alcoholic Fatty Liver Disease (NAFLD) and Triple-Negative Breast Cancer (TNBC)

The article titled “Identification of Hub Genes and Biological Mechanisms Associated with Non-Alcoholic Fatty Liver Disease and Triple-Negative Breast Cancer” identified hub genes associated with NAFLD and TNBC by analyzing publicly available transcriptomic data [2]. This study also explored the potential co-pathogenesis and prognostic linkage between these two diseases.

### 1.4. Breast Cancer Exposomics

The review titled “Breast Cancer Exposomics” discusses the impact of environmental exposures on the development of breast cancer, including the roles of environmental toxins, dietary components, psychosocial stressors, and their associated biological processes and molecular pathways [7]. This review emphasized the role of food and nutrition, as well as endocrine-disrupting chemicals (EDCs), in breast cancer development.

### 1.5. The Foundational Role of Breast Cancer Cell Lines in Cancer Research

“Molecular, Cellular, and Technical Aspects of Breast Cancer Cell Lines as a Foundational Tool in Cancer Research” reviews the history and origins of breast cancer cell lines and analyzes the molecular pathways that pharmaceutical drugs apply to these cell lines in vitro and in vivo [8]. This review also discussed controversies regarding the use of patient-derived xenografts (PDXs) versus cell-derived xenograft (CDXs) and 2D versus 3D cell culturing techniques.

### 1.6. Progress and Challenges of Immunotherapy Predictive Biomarkers for Triple-Negative Breast Cancer

“Progress and Challenges of Immunotherapy Predictive Biomarkers for Triple Negative Breast Cancer in the Era of Single-Cell Multi-Omics” discusses the advancements in single-cell sequencing techniques that have allowed for a deeper exploration of the complex and heterogeneous TNBC tumor microenvironment [4]. This review highlighted the potential of single-cell multi-omics analysis for identifying more effective biomarkers and personalized treatment strategies for TNBC patients.

### 1.7. Adverse Events of PD-1 or PD-L1 Inhibitors in Triple-Negative Breast Cancer

“Adverse Events of PD-1 or PD-L1 Inhibitors in Triple-Negative Breast Cancer: A Systematic Review and Meta-Analysis” provides a comprehensive understanding of treatment-related adverse events when using PD-1 or PD-L1 inhibitors in TNBC [9]. This study included an analysis of the incidence of serious immune-related adverse events and suggested considerations for their management.

### 1.8. In Silico Analysis of Triple-Negative Breast Cancer-Specific Biomarkers

“In Silico Analysis of Publicly Available Transcriptomic Data for the Identification of Triple-Negative Breast Cancer-Specific Biomarkers” employed in silico analyses to identify biomarkers for triple-negative breast cancer (TNBC), a subtype with limited treatment options [10]. Using publicly available transcriptomic data, the researchers of this study identified 34 differentially expressed genes (DEGs) associated with TNBC. These findings could help in developing targeted therapies and improving diagnostic accuracy.

### 1.9. Neuroendocrine Breast Carcinoma: Characteristics and Prognosis

“Clinicopathological Characteristics and Prognostic Profiles of Breast Carcinoma with Neuroendocrine Features” examined the clinicopathological characteristics and prognostic outcomes of breast carcinoma with neuroendocrine features [1]. This study found that these tumors are generally hormone receptor-positive and have a higher prevalence among postmenopausal women. Factors such as diabetes and advanced disease stage were associated with poorer progression-free survival.

### 1.10. Advancements in Post-Mastectomy Breast Reconstruction

“Breast Reconstruction following Mastectomy for Breast Cancer or Prophylactic Mastectomy: Therapeutic Options and Results” discusses various reconstructive options following mastectomy for breast cancer or as a preventive measure [11]. It highlights the evolution of techniques and materials that offer women more choices for breast restoration, aiming to improve psychological outcomes and quality of life after surgery.

### 1.11. Artificial Intelligence in Breast Cancer Diagnosis: Patient Perspectives

“Patients’ Perceptions and Attitudes to the Use of Artificial Intelligence in Breast Cancer Diagnosis: A Narrative Review” synthesizes patient perspectives on the use of artificial intelligence (AI) in breast cancer diagnostics [3]. It reveals that while there is interest in AI’s potential to improve diagnostic accuracy, there is also significant concern regarding trust and the desire for human oversight in the diagnostic process.

### 1.12. Investigating the Role of Eosinophils in Reactive Breast Stroma

“Eosinophilic Dermatoses: Cause of Non-Infectious Erythema after Volume Replacement with Diced Acellular Dermal Matrix in Breast Cancer?” explores the role of eosinophils in reactive breast stroma, particularly in the context of inflammation and tumor microenvironment interactions [12]. The findings of this study suggested that eosinophils may play a part in the breast’s response to tumor presence, although their exact role remains to be fully understood.

## 2. Advancing Frontlines: New Perspectives in Breast Cancer Research

Currently, several critical areas in breast cancer research are drawing considerable attention. Among them, significant advancements in immunotherapy, particularly for TNBC, are at the forefront [13]. TNBC is known for its aggressive nature and lack of targeted therapies, which makes the development of effective immunotherapy treatments especially crucial [14]. These treatments aim to harness the body’s immune system to better recognize and combat cancer cells, offering new hope for improving survival rates in a subgroup of breast cancer that has traditionally been challenging to treat [15]. Recent studies have highlighted the effectiveness of treatments like pembrolizumab, which, when combined with chemotherapy, has shown to improve survival rates in patients with high-risk early-stage TNBC [16,17].

Another major area of focus is the management of HER2-positive breast cancer. This subtype, characterized by the overexpression of the HER2 protein, has seen transformative treatments in recent decades, such as targeted therapies that significantly improve patient outcomes [18]. Research is ongoing to enhance these therapies’ efficacy and reduce side effects, ensuring more patients can benefit from these advanced treatments [19,20].

Additionally, the role and optimization of radiotherapy in breast cancer treatment protocols remain critical [21]. Radiotherapy is a cornerstone of breast cancer management, used both in the early and more advanced stages of this disease [22]. Innovations in radiotherapy techniques aim to increase the precision and effectiveness of radiation delivery, minimize damage to surrounding healthy tissues, and enhance its cancer-killing capabilities [23,24].

These research topics reflect a concerted and multidisciplinary effort to improve patient survival rates, manage risk factors more effectively, and refine surgical and chemotherapy strategies to offer tailored and less invasive treatment options. By pushing the boundaries in these key areas, researchers hope to not only extend the lives of those diagnosed with breast cancer but also improve their quality of life during and after treatment [25].

## 3. Final Reflections

This Special Issue embodies our collective quest to understand the complexities of breast cancer through cutting-edge research and to translate these discoveries into actionable treatments that improve patient outcomes. Through a multidisciplinary lens, we explore innovative diagnostic tools [5,10], breakthrough therapies [4,9], and pioneering surgical techniques [11] that are reshaping the way we approach this disease. Our contributors, leading experts in their fields, offer insights into the evolving paradigms of breast cancer management, from molecular genetics to personalized medicine. Their work not only reflects the current state of knowledge but also charts a course for future research directions. We invite you to delve into these pages, where the synergy of scientific discovery and clinical excellence illuminates the path toward a world with more effective breast cancer treatments.
